# Application of Stem Cell Therapy for Infertility

**DOI:** 10.3390/cells10071613

**Published:** 2021-06-28

**Authors:** Sarama Saha, Partha Roy, Cynthia Corbitt, Sham S. Kakar

**Affiliations:** 1 Department of Biochemistry, All India Institute of Medical Sciences, Rishikesh 249203, India; saramasaha@yahoo.co.in; 2 Department of Biotechnology, Indian Institute of Technology, Roorkee 247667, India; partha.roy@bt.iitr.ac.in; 3 Department of Biology, University of Louisville, Louisville, KY 40292, USA; cynthia.corbitt@louisville.edu; 4 Department of Physiology and James Graham Brown Cancer Center, University of Louisville, Louisville, KY 40292, USA

**Keywords:** infertility, mesenchymal stem cell, induced pluripotent stem cell, spermatogonial stem cell, assisted reproduction technology

## Abstract

Infertility creates an immense impact on the psychosocial wellbeing of affected couples, leading to poor quality of life. Infertility is now considered to be a global health issue affecting approximately 15% of couples worldwide. It may arise from factors related to the male (30%), including varicocele, undescended testes, testicular cancer, and azoospermia; the female (30%), including premature ovarian failure and uterine disorders; or both partners (30%). With the recent advancement in assisted reproduction technology (ART), many affected couples (80%) could find a solution. However, a substantial number of couples cannot conceive even after ART. Stem cells are now increasingly being investigated as promising alternative therapeutics in translational research of regenerative medicine. Tremendous headway has been made to understand the biology and function of stem cells. Considering the minimum ethical concern and easily available abundant resources, extensive research is being conducted on induced pluripotent stem cells (iPSCs) and mesenchymal stem cells (MSC) for their potential application in reproductive medicine, especially in cases of infertility resulting from azoospermia and premature ovarian insufficiency. However, most of these investigations have been carried out in animal models. Evolutionary divergence observed in pluripotency among animals and humans requires caution when extrapolating the data obtained from murine models to safely apply them to clinical applications in humans. Hence, more clinical trials based on larger populations need to be carried out to investigate the relevance of stem cell therapy, including its safety and efficacy, in translational infertility medicine.

## 1. Introduction

Fertility is the ability to achieve clinical pregnancy. According to the international glossary, infertility is defined as “A disease characterized by the failure to establish a clinical pregnancy after 12 months of regular, unprotected sexual intercourse or due to an impairment of a person’s capacity to reproduce either as an individual or with his/her partner.” Fertility interventions may be initiated in less than 1 year based on medical, sexual, and reproductive history, age, physical evaluation, and diagnostic testing. Infertility is a disease, which generates disability as an “impairment of function” [1]. While the term infertility delineates a condition of a restricted time period, sterility illustrates a permanent state of infertility [1]. Infertility has emerged as a global health issue [2] that may arise from factors related to male or female or both. Approximately 12–15% of reproductive-age couples, representing more than 48 million couples and approximately 72 million people, are affected by infertility worldwide [3], of which approximately 30% are exclusively contributed by male factors [4], and approximately 30% are from the combination of both partners. According to Single Care’s infertility survey, the cause of 8–28% of infertility cases remains unexplained (https://www.singlecare.com/blog/news/infertility-statistics/accessed on 30 March 2021). In developing countries, 1 in 4 couples suffers from infertility (WHO 2004). According to a CDC (2013) report and Office on Women’s Health (2019) (https://www.womenshealth.gov/a-z-topics/infertility/accessed on 30 March 2021), in the United States, approximately 9% of men and 10% of women of reproductive age experience infertility [5]. According to the Central Intelligence Agency (2017), Africa contributes to the highest brunt of infertility cases, followed by Afghanistan. On the other hand, according to the United Nations Population Fund (2018), Southern Europe and Eastern Asia have the lowest fertility rates, at 1.5 children per woman on average [6]. This statistical information emphasizes that a substantial number of couples worldwide are having trouble with conception. Hence, many couples are deprived of having genetically related offspring. Conventional treatment, including ovulation-inducing drugs, or assisted reproduction technology (ART) could resolve these issues to a certain extent. However, these existing treatments enhance the risks of multiple pregnancies or lack efficacy in some couples, both of which create a financial burden to the couple. To overcome the shortcomings of the current treatment methods and considering the ability of stem cells to replenish damaged tissues via their self-renewal properties and ability to differentiate into multiple lineages, stem cells could be considered as a novel therapeutic measure for infertility [7,8]. Extensive preclinical and clinical trials are in the pipeline to investigate the safety and efficacy of stem cells as another promising therapeutic approach for the restoration of fertility [9].

## 2. Infertility Risk Factors

Numerous risk factors have been reported to contribute to infertility, the most common being the woman’s age. Hence, evaluation of infertility may be started after 6 months if the female partner’s age exceeds 35 years and unable to conceive, whereas an immediate evaluation is recommended if the woman’s age exceeds 40 years. However, other potential attributing factors need to be evaluated to narrow down the differential diagnosis to establish a more specific and targeted therapeutic approach. To accomplish this, detailed historical evaluations should be carried out related to sexually transmitted diseases (such as Chlamydia), pelvic inflammatory diseases, smoking, alcohol consumption, exposure to environmental toxins (such as lead), and pesticides that are associated with risk factors for infertility in both genders. In addition, metabolic disorders such as obesity, polycystic ovarian syndrome (PCOS), and diabetes mellitus may lead to anovulation and increase the prevalence of infertility in the affected couple [10].

Some factors that solely contribute to female infertility include abnormal menstruation, history of tubal pregnancy, ovulatory disorders, and uterine abnormalities, which include endometriosis, uterine fibroids, uterine polyps, and intrauterine adhesion (Asherman syndrome) [7]. According to WHO, ovulatory disorders are divided into three categories: i. low body weight, female presenting with amenorrhoea, and decreased gonadotropins levels resulting from hypothalamic-pituitary failure; ii. PCOS that is observed in 85% of the cases and hyperprolactinemia resulting from hypothalamic pituitary ovarian axis dysfunction; and iii. ovarian failure is observed only in 5% of women who achieve pregnancy only after oocyte donation and in vitro fertilization [11].

Similarly, there are several causes that are purely responsible for male infertility, and these include hypogonadism (small gonads resulting in low circulating levels of testosterone), injury to the testicles, premature ejaculation, varicocele, undescended testicles, testicular cancer, and azoospermia [12,13]. Overall, male factors contribute to approximately half of the infertility cases, and nonobstructive azoospermia is the most common factor in males [14].

**Figure 1:**
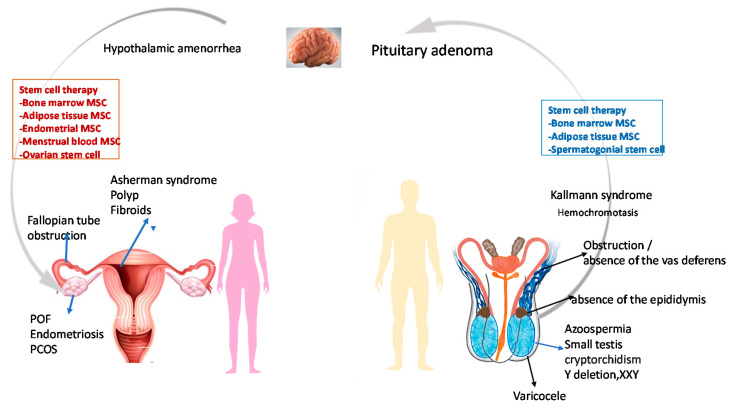
Some probable causes of infertility in male and female POF: premature ovarian failure, PCOS: Polycystic ovarian syndrome, XXY: Klinefelter syndrome, MSC: Mesenchymal stem cell. Modified from references Lindsay and Virtikas [8] and Zhao et al. [11].

The other common factors contributing to infertility include genetic disorders such as fragile X syndrome, Turner syndrome, cystic fibrosis, Klinefelter syndrome, deletion of the azoospermia factor c region on the Y chromosome, mutations in androgen receptor (AR), and the presence of a CAG triplet repeat expansion in AR [15,16]. Causes of infertility are summarized in Figure 1.

Infertility is often considered a social stigma and can create psychological trauma to the couple, hence becoming a stressful experience for both partners while they try to achieve pregnancy. Surprisingly, when a couple is unable to conceive, the social stigma associated with infertility is primarily suffered by the female partner. Anxiety over impaired ability to conceive compounds the stress and further declines libido. Therefore, formal and early counselling could be helpful for couples who are experiencing difficulty in conceiving [17] in order to initiate treatment at the earliest possible time [18].

## 3. Conventional Treatment

Conventional treatments improve fertilization rates inside the womb. Conventional treatments for male infertility are mainly focused on improving sperm quality, which depends on etiologic factors. In the case of endocrinopathy, such as hyperprolactinemia, the cause is identified and treated accordingly. In the case of varicocele, a surgical correction is carried out [19]. Evidence-based medicine revealed a significant improvement in pregnancy rate after treatment with antioestrogens and gonadotropins therapy in cases of idiopathic male infertility [20]. Administration of antioxidants such as vitamin E and zinc along with assisted reproduction technology enhanced the rate of live offspring in infertile couples [21,22]. 

Various conventional techniques are presently used to overcome female infertility due to ovulatory failure. Application of ovulation-inducing drugs depends on the type of anovulatory disorder; for example, in the case of hypogonadotropic hypogonadism, gonadotropins and pulsatile gonadotropin-releasing hormone (GnRH) therapy is preferred. Previously, human menopausal gonadotropins, a mixture of follicle-stimulating hormone (FSH) and luteinizing hormone (LH), were used. However, at present, more expensive but purer forms of recombinant gonadotropins are recommended. On the other hand, in the case of eugonadotropic eugonadism in which patients present with PCOS, Clomiphene citrate and letrozole are drugs of choice [23]. Clomiphene citrate increases FSH level through its antiestrogenic action and blockage of estrogen receptors at the pituitary gland and thus promotes multiple follicular growths. Letrozole is an aromatase inhibitor and thus inhibits the conversion of androgen to estrogen, resulting in increased FSH production from the pituitary gland. The risk of multiple pregnancies with injectable gonadotropins is much higher (30%) as compared to orally administered clomiphene citrate or letrozole (7%). In cases of hyperprolactinemia, bromocriptine, or cabergoline is used to improve fertility indirectly through the normalization of prolactin levels [24]. After the administration of ovulation-inducing drugs, regular follicular monitoring is carried out using ultrasonography in order to identify the time of formation of the dominant follicle (18–20 mm), which would be followed by ovulation trigger by intramuscular injection of βhCG [25].

Intrauterine insemination (IUI) is a conventional treatment used for both male and female infertility. After collection of semen from the male partner, highly motile sperm with normal morphology are isolated. The highest quality isolated sperm are injected within the uterine cavity with the help of a thin malleable catheter bypassing the cervix approximately 36 h after natural ovulation or triggered ovulation [25].

## 4. Assisted Reproduction Technology (ART)

In contrast to conventional treatments that improve fertilization rates inside the womb, assisted reproductive technologies (ARTs) are defined as therapeutic approaches for infertility that involve the handling of either eggs or embryos. ART includes several steps, including surgical removal of eggs followed by fertilization with sperm in the laboratory and transferring fertilized embryos into the woman’s womb or preserving them for donation to other women. In the past, ART was used only for problems related to fallopian tubes such as blockage, damage, or absence. However, at present, this technology is also used for other conditions such as severe male factor infertility, treatment failure with ovulation induction alone or in combination with IUI, unexplained infertility, for donor oocytes in case of premature ovarian insufficiency (POI), for surrogacy in cases of uterine problems such as Asherman syndrome, or to perform a preimplantation genetic diagnosis to exclude chances of transmission of any genetic disorder to the progeny. Various steps involved in ART include:

### 4.1. Ovarian Stimulation

To increase the success rate of fertilization and subsequent live birth, multiple eggs are stimulated to grow instead of the development of a single egg that happens naturally every month. Drugs commonly used for super-ovulation include Clomiphene citrate, Letrozole, human chorionic gonadotropin, luteinizing hormone (LH) in combination with FSH, and human menopausal gonadotropin. Clomiphene citrate and letrozole are administered orally and are less efficient compared to other injectable drugs and hence are not commonly used in ART. An ART cycle is started with a hormonal regimen of GnRH agonist followed by FSH and human menopausal gonadotropin for the growth and development of multiple follicles [26]. Timing is the primary determinant for successful oocyte retrievals and subsequent steps of ART. Hence, the response to ovulation-inducing drugs and regular monitoring of the development of ovarian follicles is carried out with the help of measurement of blood levels of estrogen and progesterone and vaginal ultrasound examinations, respectively. Responsiveness to ovulation-inducing drugs is decreased with the age of the female partner, especially after 40 years [27].

### 4.2. Oocyte Retrieval

In oocyte retrieval, eggs are aspirated 34–36 h after administration of hCG through the vaginal fornix with the help of an ultrasound-guided needle attached to a suction device [28]. If the ovaries are not accessible through the vagina, eggs are collected with the help of laparoscopy. Immediately after aspiration, oocytes are washed in a petri dish containing culture media and examined under a microscope to identify the stage of meiosis in the eggs based on their intracellular and extracellular composition [26].

### 4.3. In Vitro Fertilization and Embryo Culture

Immediately after the microscopic examination, the eggs are cultured in media that has a similar composition as fallopian tubes for approximately 6 h and then allowed to get inseminated. Approximately 20 h after insemination, eggs are examined for signs of fertilization, such as documentation of two pronuclei and polar bodies [29]. However, in cases of couples having male factor infertility and seeking ART intervention, intracytoplasmic sperm injection (ICSI) in which a sperm is injected directly into an egg is preferred to facilitate sperm penetration and thus increases the success rate of having live birth [30]. In this way, approximately 65–75% of mature oocytes get fertilized [31]. However, this fertilization rate is decreased in cases of poor quality of sperm or eggs. After the egg starts dividing, by the third day, the embryo contains 6–10 cells. By the fifth day, the embryo develops a fluid cavity, and at this stage, it is known as blastocyst [26].

### 4.4. Embryo Transfer

One or more developing embryos between 1 and 6 days (preferably blastocyst stage) suspended in culture media are transferred into the uterus via a catheter attached with a syringe on one end. The maximum number of embryos to be transferred is determined by the balance of patient age, the status of the embryos, and the risk of multiple pregnancies. Subsequently, the developing embryos undergo implantation into the lining of the endometrial cavity. Excess embryos after transfer may be cryo-preserved for future purposes. Depending on the developmental stage and departmental skill, embryo cryo-preservation may be performed in two ways, conventional or slow freezing and “vitrification” or fast freezing.

In the case of older women or any couple with a previous history of IVF failure, a micromanipulation technique to enhance the hatching of the developing embryo through digging a hole into the zona pellucida just prior to the transfer of the embryo, assisted hatching (AH), can be employed [32]. Although some randomized trials documented that there is no beneficial role of AH in enhancing live birth rates [33], a meta-analysis found that assisted hatching could enhance the live birth rate, especially in cases of previous IVF failure [34].

## 5. Implications for Stem Cells in Infertility

Even after recent progress in ART, many couples are unable to parent healthy babies except through gamete donation or adoption. Infertility due to gamete deficiency resulting from genetic defects does not benefit from ART. However, most couples seeking infertility treatment wish to have their own genetically related issues resolved [35], which could be less invasive and more cost-effective compared to ART. In this respect, stem cells have shown new hope to overcome the issues related to infertility in the form of cell-based therapies in various experimental preclinical and clinical models [36,37,38,39,40,41,42,43,44].

Stem cells are a subtype of cells that remain in undifferentiated form in embryos and in adult tissues and can self-renew and differentiate as and when required. Stem cells in differentiated organs contribute to the restoration of function through organ damage repair. According to their origin, stem cells are classified as embryonic stem cells (ESC), adult stem cells (includes mesenchymal stem cells MSC), induced pluripotent stem cells (iPSC), spermatogonial stem cells (SSCs) [45], and ovarian stem cells [46]. Stem cells collected from various organs explored to treat infertility are listed below: 

### 5.1. Embryonic Stem Cells (ESC)

ESCs play an important role in regenerative medicine because of their indefinite capacity to self-renew, ability to differentiate into all three lineages (ectoderm, endoderm, and mesoderm) and ability to maintain the normal karyotype during growth. Human embryonic stem cells (hESC) are derived from the inner cell mass of blastocysts and express transcription factor Oct 4 [47]. It was documented that both mouse and human ESC can differentiate into primordial germ cells in vitro and subsequently undergo meiosis and give rise to male and female gametes [48]. Yuan et al. [49] documented in a sperm-deficient mice model (Kit^w^/Kit^wv^) the feasibility of developing functional sperm by using gene repaired ESC isolated from cloned blastocysts that were originated from nuclear transferred somatic cells (ntESC) using gene repair technology [49]. Hence, ESCs have emerged as an important tool for cell-based therapy to address the issues of infertility. However, because of ethical concerns, even after initial derivation, it is not so commonly used in cell replacement therapy. The hESCs have been documented to play a crucial role in damage repair and restoration of function of the endometrium [45].

### 5.2. Induced Pluripotent Stem Cells (iPSC)

In 2006, Takahashi and Yamanaka [50] revolutionized cell-based therapy with the induction of pluripotent stem cells from mouse fibroblast culture with the help of four transcription factors: Oct4, klf4, sox2, and c-myc. They also observed that iPSCs generated after reprogramming resemble ESCs with respect to morphology, expression of surface markers, telomerase activity, the capacity of differentiation to all three lineages and having normal karyotype for growth. The iPSCs are superior to ESCs in regenerative medicine because they originate from adult cells, thereby avoiding the ethical issues of using embryos, and they are readily available. Moreover, iPSCs are developed from the patients’ own somatic cells, and hence, there is less chance of immune rejection [35,51]. Various studies have been conducted for the differentiation of iPSCs to male germ cells in vitro. Eguizabal et al. [52] differentiated keratinocytes and cord blood to haploid gamete-like cells. To achieve differentiation, they initially used retinoic acid (RA) in the culture medium for 3 weeks. Next, they harvested the cells in the presence of forskolin, human recombinant leukemia inhibiting factor (LIF), and the CYP26 inhibitor R115866 for 2, 3, and 4 more weeks [52]. Ramathal et al. [53] cultured human skin cells derived from men with azoospermia and fertile men in the presence of BMP4, BMP8, RA, LIF, and then xenotransplanted them into the testes of immune-deficient nude mice generated by busulfan treatment. They documented that iPSCs transplanted into seminiferous tubules differentiated into germ cell-like cells (GCLCs), whereas cells outside the tubule could not result in GCLCs. Sperm and ova are derived from primordial germ cells (PGCs) [53]. Irie et al. [54] documented that SOX17 was the critical regulator of human PGCs, whereas BLIMP1 acted as a repressor during specification. In their study, they used Fragile X male and female patient-derived iPSCs. Sasaki and colleagues [55] documented that human iPSCs could differentiate into hPGCs in the presence of Activin A, CHIRON, BMP4, SCF, EGF, and LIF. They isolated the human PGCs from other cells with the help of markers present on PGCs such as EpCAM and Integrinα6 [55]. Other studies also reported the differentiation of the fibroblast-derived iPSCs into spermatogenic cells either in a normal culture medium or in vivo xenotransplantation technique [56].

**Figure 2:**
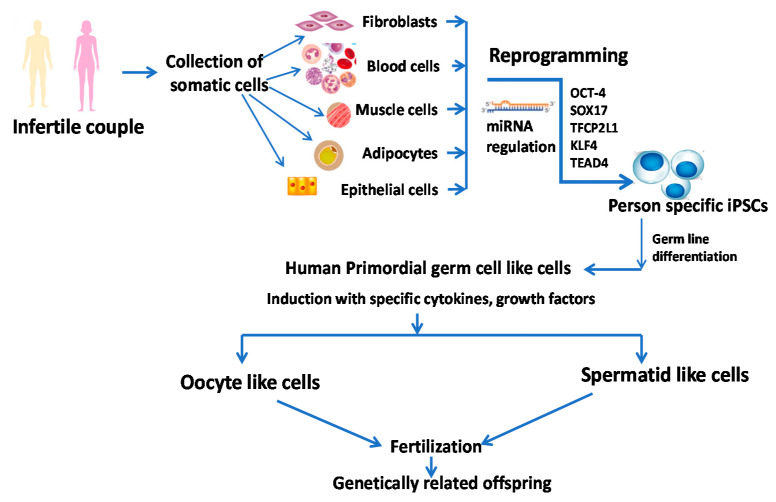
The role of stem cells in the generation of patient-specific gamete cells. TEAD4: trophectoderm regulator TEA domain transcription factor 4; SOX17: endoderm regulator SRY-box 17; TFCP2L1: pluripotency factors transcription factor CP2-like 1; KLF4: Kruppel-like factor 4; miRNA: Micro RNA; OCT-4: octamer-binding transcription factor 4. Modified from references [35,57,60].

Yamashiro et al. [57] and Gell and Clark [58] reported the feasibility of differentiation of iPSCs into oogonia giving hope to couples with poor ovarian reserve or inability to develop functional eggs. In their studies, at first, they differentiated somatic cells into iPSC by reprogramming and then generated incipient mesoderm-like cells (iMeLCs) in the presence of Activin A and Chiron. This was followed by the generation of human primordial germ cell-like cells (hPGCLCs) by plating iMeLCs in aggregates. These hPGCLCs were mixed with gonadal cells isolated from female mouse embryonic ovaries and cultured for 4 months to obtain oogonia by in vitro gametogenesis. This technique will bring a revolution in reproductive medicine through the generation of oogonia from human pluripotent stem cells. Sugawa et al. [59], on the other hand, documented the mechanistic explanation of differentiation of human pluripotent stem cells to heterogeneous mesoderm-like cells in the presence of cytokines followed by the generation of PGC-like cells (PGCLCs), and the PGCLCs are committed to developing gametes (sperm or eggs) that were associated with a unique expression of PRDM14 [59]. Generation and proposed therapeutic implications of iPSCs in infertility are presented in Figure 2. Sources: [35,57,60].

In spite of promising results obtained from experimental models, the teratogenic potentiality of iPSCs and their derivatives resulting from reprogramming by induction with oncogenes (for example, c-myc) and use of nucleic acid integration procedures restrict their clinical application towards personalized cell-based therapy [61]. Moreover, the persistence of residual epigenetic impressions and gene silencing, as well as genomic instability, might be a potential hurdle that further limits their therapeutic application [62,63].

Although the iPSC technology does not destroy the embryos, the potentiality to exploit the embryo generated from gametes developed after iPSCs reprogramming demand ethical clearance from the institutional review board as well as the collection of informed consent from the cells or tissue donor prior to acquiring any sample for development of iPSC for clinical trials as well as research purpose. In addition, for their application in animal models, approval is required from IACUC.

### 5.3. Mesenchymal Stem Cells (MSCs)

According to the mesenchymal and tissue stem cell committee of the International Society for Cellular Therapy, MSCs are defined as cells that have plastic-adhesion properties, express CD105, CD73 and CD90 as surface markers, and can differentiate into osteoblasts, adipocytes and chondroblasts [64]. Depending on their origin, MSCs are categorized as bone marrow stromal cells, adipose-derived stem cells, menstrual-blood-derived MSCs and umbilical-cord-derived MSCs [8], amniotic-fluid-derived MSCs, placental-tissue-derived MSCs, salivary-gland-derived MSCs, and dental-pulp-derived MSCs [7]. Various preclinical and clinical studies have reported the potential therapeutic application of MSCs for the treatment of infertility due to ovarian and endometrial dysfunction [65,66,67]. MSCs travel to the damaged ovary and settle there to restore ovarian function through the release of various cytokines by paracrine mechanisms. Cytokines such as vascular endothelial growth factor, insulin-like growth factor, and hepatocyte growth factor induce the formation of new vessels, prevent apoptosis as well as fibrosis and thus ameliorate ovarian dysfunction. MSCs also help in the revival of fertility by endometrial regeneration through the release of various bioactive molecules that modulate inflammation and other immune reactions and activate tissue-specific progenitor cells [8].

Adult MSCs are found to have reduced potency and abundance, decreased rate of multiplication and ability to differentiate into multiple lineages with age [68]. This propensity of senescence and variation in potency might limit the desired therapeutic outcome in all cases. On the contrary, fetal subpopulations of MSCs exhibit a higher proliferation rate leading to rapid expansion and efficient differentiation to multilineages as well as strong immunomodulatory properties [69]. Augmented telomerase activity might contribute to better performance and prolonged survival of the fetal MSCs (FMSC) compared to adult MSCs [69]. Fetal MSCs can also be demonstrated in the blood, bone marrow, and liver during the first trimester. Moreover, FMSCs might also be isolated from extra embryonic tissues such as human umbilical cord blood, Wharton’s Jelly, amnion and placenta [69]. FMSCs express MSC phenotypes such as CD105, CD29, and CD90, as well as pluripotent stem cell markers such as Oct-4, Nanog, and Rex-1 [70]. Fetal and adult MSCs follow different signaling pathways to execute their functions [71]. Huang et al. [72] documented that FMSCs could ameliorate ovarian follicular function in a cyclophosphamide-induced mice model via modulation of melatonin membrane receptor 1 and antioxidant enzyme activity. Some of the sources used to derive MSCs used for the treatment of infertility are listed below:

#### 5.3.1. Bone Marrow Mesenchymal Stem Cells (BMSC)

After its initial documentation in 1988 by Owen and Friedenstein [73], the isolation of MSCs from bone marrow was carried out by density gradient centrifugation and then incubated in a growth medium for expansion [74]. These BMSCs are not only differentiated to mesodermal lineages but also committed to the development of endometrial and follicular cells in rat models [75,76].

Jing et al. [77] explored whether BMSCs could contribute to endometrial regeneration in the experimental rat model. They transplanted BMSCs through rat tail intravenous injection and observed a significant increase in thickness of endometrium along with enhanced expression of various markers, cytokeratin, vimentin, integrin αγβ3, and leukemia inhibitor factor. The constructive role of BMSCs was probably due to its homing in on the target and immune-modulatory properties manifested by upregulation of anti-inflammatory cytokines such as fibroblast growth factor-β (bFGF-β) and interleukin-6 (IL-6), and downregulation of proinflammatory cytokines, tumor necrosis factor-α (TNF-α), and interleukin-1β [77]. Later, in 2016, Wang et al. [78] endorsed the therapeutic implication of BMSCs in endometrial repairing. In an experimental animal model, intrauterine adhesion was induced by mechanical injury, and BMSCs were injected either through a tail vein or directly into the uterus. It was observed that in both cases, BMSCs could effectively heal the endometrial injury, as evidenced by the increase in the number of endometrial glands and decrease in the fibrotic area through upregulation of estrogen and progesterone receptor expression [78]. Chemotherapy to cancer patients not only destroys cancer cells but also induces programmed death of granulosa cells that play a crucial role in providing nourishment to eggs through the release of estrogen and other paracrine factors, giving rise to ovarian failure. In 2013, Abd-Allah and co-workers reported the therapeutic application of BMSCs for cyclophosphamide-mediated ovarian dysfunction in a rabbit model and explored the underlying mechanism. They reported that intravenous injection of BMSCs could restore the structure and function of ovarian follicles through downregulation of FSH and upregulation of estrogen and VEGF levels [36]. Badawy, in 2017, carried out a similar experiment on a white mice model and documented that 21 days after administration of BMSCs, there was a revival of hormonal levels and pregnancy in infertile mice. Santamaria et al. [38] conducted a prospective, experimental pilot study on patients with Asherman’s syndrome/endometrial atrophy and reported that injection of CD133+ BMSCs into the spiral arterioles of recruited patients could improve endometrial thickness, the intensity of matured blood vessels, and the nature of menstruation of infertile patients. Moreover, 10 out of 16 patients conceived either spontaneously or after embryo transfer [38]. Altogether, these studies provide evidence that BMSCs could open new hope for having biological offspring in cases of ovarian or uterine dysfunction.

#### 5.3.2. Menstrual Blood Mesenchymal Stem Cell (MB-MSC)

Menstrual-blood-derived MSCs (MB-MSCs) have the potential to proliferate and differentiate into multiple lineages and can self-renew similar to other stem cells. Moreover, the collection of these cells is noninvasive, safe, and easy, without ethical issues and with minimal immune reactions, facilitating their clinical application in reproductive medicine compared to other tissue-derived stem cells.

Liu et al. [79] reported the impact of MB-MSCs on cyclophosphamide-induced premature ovarian failure (POF) in a mouse model. They have shown that MB-MSC treated groups of animals showed an increase in the number of the normal ovarian follicles and restoration of normal ovarian function represented by a higher level of ovarian hormones such as estradiol, antimullerian hormone and inhibin α/β compared to control. Manshadi et al. [39] conducted similar experiments on the busulfan-induced rat model of POF. In their studies, they documented that MB-MSC improved ovarian function through localization of MSCs into granulosa cells and by augmentation of the expression of FSH receptors as well as restoration of hormone levels [39]. It was documented that MSC derived from menstrual blood could resume fertility in an animal model of damaged endometrium through induction of angiogenesis and release of anti-inflammatory factors [40]. Zheng et al. [80] documented for the first time a theoretical basis of the use of MB-MSCs for the treatment of intrauterine adhesion. MB-MSCs could differentiate into endometrial cells in vitro, and subsequent transplantation in vivo to NOD-SCID mice resulted in regeneration of endometrium. The differentiation ability of MB-MSCs is contributed by the presence of the OCT-4 transcription factor. In another study, Zhang and his colleagues [81] also documented that MB-MSCs along with platelet-rich plasma could efficiently induce resumption of fertility in a mechanical injury-induced rat model of intrauterine adhesion through significant modulation of the Hippo signalling pathway and its downstream regulators such as CTGF, Wnt5a, and Gdf5 [81].

Moreover, MB-MSCs could improve epirubicin (broad-spectrum anti-cancer drug)-induced ovarian damage and thus promote the multiplication of granulosa cells through upregulation of Cyclin B1 and CDC2 protein expression and downregulation of Gadd45b protein expression [82].

A clinical study conducted by Tan et al. [83] reported on seven patients with resistant Asherman syndrome treated with MB-MSC transplantation followed by hormonal therapy; out of these seven patients, five patients attained endometrial thickness of 7 mm, one out of these five patients achieved pregnancy spontaneously and two conceived after assisted reproduction technology [82]. The role of the OCT-4 transcription factor on differentiation of MSCs documented in animal models was supported by the decreased expression of OCT-4 observed in MB-MSCs isolated from patients with severe intrauterine adhesion [80].

#### 5.3.3. Endometrial Stem Cells (EndSCs)

Following the initial introduction of endometrial stem cells by Prianishnikov and his colleagues in 1978 [84], Chan and his colleagues in 2004 [85] isolated stem cells from endometrial tissues and identified the colonization properties of these cells in vitro. Later, Gargett and coworkers [86] isolated and cultured MSCs from human endometrium based on their expressed markers and speculated on their contributions in endometriosis and endometrial cancer [86]. The EndSC microenvironment is comprised of stromal cells, epithelial progenitor cells and endothelial cells. Stromal cells were isolated by the presence of CD44, CD73, CD90, and the absence of CD34 and CD45 phenotypes, while epithelial progenitor cells demonstrated the presence of stage-specific embryonic antigen (SSEA-1), N-cadherin, and NTDPase2 activity. However, endothelial stem cells documented the presence of a classical phenotype such as CD 31 and CD 34 [9]. In the absence of any injury, these stem cells were present in quiescent undifferentiated form within the endometrium. However, in the presence of inflammation or damage to the endometrium, these stem cells were directed to the injured site following interaction between chemokines and CXCR4, which is expressed on stem cells, and CXCL12, which is expressed by the epithelium of endometrial tissues [87]. Endometrial stem cells act as the origin of cells that help in replenishing the endometrium, such as mesenchymal stem cells (MSCs), epithelial stem cells, endometrial side population cells (ESP), and endometrial regenerative cells (ERC) [88]. ESPs constitute only 5% of EndSCs and are primarily present in the proliferative phase of the menstrual cycle [89]. ERCs such as MSCs can undergo differentiation into multiple tissues such as adipose tissue, neuronal tissue, bones, and cartilage [90]. They can induce immune regulation, angiogenesis as well as the release of matrix metalloproteinases more efficiently compared to MSCs. Appropriate blood circulation in the uterus is required for the maintenance of different stages of reproduction, including the development of the endometrium [91]. It was observed that inadequate neovascularization probably due to the impaired release of vascular endothelial growth factor resulted in thinning of endometrium in an animal transplantation model [92]. Hence, this angiogenic property of endometrial stem cells could be implicated in the therapeutic transplantation of reproductive medicine for endometrial regeneration. Endometrial stem cells can also be derived from menstrual blood.

**Figure 3:**
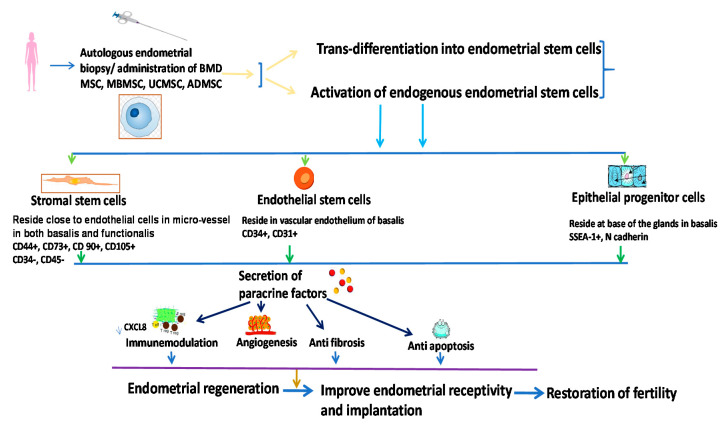
The mechanistic explanation of stem cell therapy for restoration of fertility via endometrial regeneration; BMDMSC: Bone-marrow-derived mesenchymal stem cell; MB MSC: menstrual blood mesenchymal stem cell; UC MSC: umbilical cord mesenchymal stem cell; ADMSC: adipose-tissue-derived mesenchymal stem cell; CXCL: C-X-C motif chemokine ligand; SSEA-1: Stage-specific embryonic antigen-1; CD: Cluster of differentiation. Modified from references [9,87].

Therapeutic implications of endometrial stem cells have been documented in a 1-methyl-4-phenyl-1,2,3,6-tetrahydropyridine (MPTP)-induced model of Parkinson’s Disease [93] to observe if it can restore dopamine release and in a murine model of diabetes mellitus [94]. Mechanistic explanations and roles of endometrial stem cells in the restoration of fertility are summarized in Figure 3. Modified from references [9,88].

#### 5.3.4. Umbilical Cord Mesenchymal Stem Cells (UC-MSCs)

Wharton’s jelly of umbilical-cord-derived MSCs showed the presence of CD29, CD44, CD73, CD90, and CD105 expression, with the absence of CD31, CD45, and HLA-DR85. Because of low tumorigenicity, rapid self-renewal capacity committed to various mesodermal cell types, easy availability from usually-discarded resources, minimum ethical concerns, and low immunogenicity, UC-MSCs have emerged as a popular cell-based therapeutic approach for restoration of fertility [8].

In various experimental animal models of premature ovarian insufficiency, UC-MSCs restored ovarian function through its antiapoptotic activity against granulosa cells and modulation of hormone levels, for example, reducing FSH levels while augmenting estrogen and progesterone levels [95,96]. UC-MSCs could restore ovarian function in paclitaxel-induced ovarian dysfunction through the provocation of ovarian surface epithelium stability and modulation of the expression of Cyto Keratin 8/18, TGF-ß, and proliferating cell nuclear antigen (PCNA), which are critical regulators of follicular synthesis and the antiapoptotic activity of UC-MSCs [97]. UC-MSCs also emerged to be beneficial in the revival of infertility in a mouse model of PCOS induced by dehydroepiandrosterone via inactivation of proinflammatory cytokines, for instance, IL- 1β, tumor necrosis factor alpha (TNFα), and interferon gamma (IFN-γ) [98].

The beneficial effect of UC-MSCs in the improvement of fertility might involve various signaling pathways. UC-MSCs prevent apoptosis of granulosa cells to improve fertility via the activation of mitogen-activated protein kinase signaling pathway, G protein-coupled receptor, and insulin signaling pathways [99]. UC-MSCs could resolve into endometrial cells [100]. UC-MSCs restored fertility in an animal model of damaged endometrium through the increase and decrease in vascular and inflammatory factors, respectively [101]. Similarly, transplantation of UC-MSCs on a collagen scaffold in a rat model of uterine injury could rescue fertility via activation of matrix metalloproteinase 9 [102].

Administration of UC-MSCs on a collagen scaffold could restore ovarian function in patients suffering from premature ovulatory failure (POF) as confirmed by the increase in follicle number through activation of primordial follicles via phosphorylation of transcription factor forkhead box protein O3a (FOXO3a) and FOXO1 [103]. Yang et al. [43] documented that Wharton’s jelly-derived MSC could improve proliferation and prevent apoptosis of mifepristone-induced damage in endometrium collected from patients undergoing hysterectomy via upregulation of VEGF expression and downregulation of caspase 3 and 8 [43]. Later, in 2017, Fan et al. [48] conducted a single-center, prospective, randomized, double-blind, placebo-controlled phase II clinical trial and observed the safety and efficacy of intramuscular injection of UC-MSCs for the treatment of uterine scars caused by caesarean section.

#### 5.3.5. Amniotic Fluid Stem Cells (AFSCs)

Amniotic fluid, which provides nutritional support to the fetus, acts as an important source of mesenchymal stem cells. Their immune regulatory properties, lack of ethical concern, plus ability to differentiate into various cell types such as osteocytes, muscle, and adipocytes make the AFSCs highly useful for regenerative medicine. Various animal studies have documented the therapeutic role of AFSCs in muscular and bone-related disorders [104,105]. Previous studies revealed that AFSCs could ameliorate infertility through the restoration of ovarian function via the activation of various signaling molecules such as VEGF, TGFα and β, epidermal growth factor (EGF), and bone morphogenic protein (BMP) [99]. Various studies on a mouse model of POF induced by chemotherapy documented the role of AFSCs, especially having CD4C/CD105+ markers, on the prevention of follicular atresia, reestablishment of healthy oocytes, and thus preserving ovarian function [106,107].

#### 5.3.6. Amnion-Derived Mesenchymal Stem Cells (AmDMSCs)

Amnion acts as a promising source of cell-based therapy in reproductive medicine. The restorative effect of ADMSCs on ovarian dysfunction in chemotherapy-mediated animal models of POF has been documented in various studies [108,109]. AmDMSCs restore ovarian function by attenuating apoptosis and improving the multiplication of granulosa cells and neovascularization in the surrounding environment to some extent by paracrine mechanisms. Moreover, AmDMSC treatment accomplished its function via suppression of inflammation by inactivating cytokines IL-1β, IL-6 and TNFα. Ling et al. [110] documented that surprisingly, human ADMSCs, after treatment with low-intensity pulsed ultrasound, become more efficient in the revival of ovarian function in a rat model of POF. 

#### 5.3.7. Placenta-Derived Mesenchymal Stem Cells (PDMSCs)

Various studies conducted on an animal model of POF revealed that PDMSCs produced their beneficial effects on folliculogenesis through modulation of cytokines and various hormone levels such as estradiol, LH, AMH, and FSH and their receptors’ expression by activation of PI3K/Akt signaling pathway [111,112]. On the other hand, Li et al. [113] reported the involvement of the inositol-requiring enzyme 1 (IRE1)α signaling pathway in PDMSC improved ovarian dysfunction [113]. Kim et al. [114] demonstrated in ovariectomized rats that PDMSCs are more effective in 3D spheroid formation to produce their restorative function [114]. Upregulation of miR222 could influence the differentiation of PDMSC [115].

#### 5.3.8. Adipose-Tissue-Derived Stem Cells (AD MSC)

ADMSCs are a novel type of MSCs having almost similar biological properties to MSC derived from other sources but having more advantages compared to BMSC because such tissues can be collected with less invasive procedures and in larger quantity [116]. Therefore, these are widely used for a broad spectrum of clinical conditions in reparative medicine [117,118].

One study observed the impact of ADMSCs on ovarian grafts in a female animal model and revealed that ADMSCs restored ovarian function faster through augmentation of VEGF gene expression and promotion of angiogenesis in the grafted tissue [119]. ADMSCs could restore ovarian function in chemotherapy-mediated ovarian damage in a mouse model through neovascularization and promotion of ovarian follicular proliferation [120]. However, administration of ADMSCs on a collagen scaffold in an ovarian insufficiency rat model revealed better retention of ADMSCs, giving rise to the longer resumption of ovarian function and better pregnancy rate compared to ADMSC injection alone [121]. In an experimental animal model of Asherman syndrome, ADMSCs in combination with hormone therapy could successfully reduce fibrosis and induce endometrial regeneration through angiogenesis [122]. Hence, ADMSCs could be considered an effective alternative therapeutic tool for the restoration of fertility for infertile couples, depicted in Figure 3.

### 5.4. Ovarian Stem Cells (OSC)

The invention in findings of the rate of follicular atresia along with the death of oocytes and depletion of ovarian reserve in mice brought the concept of the existence of OSCs. Johnson et al. [123] isolated these actively proliferating cells based on their ability to incorporate 5-bromodeoxyuridine (BrdU) and expression of a germ-cell specific marker, MVH (mouse vasa homologue). In their studies, they demonstrated that these mitotically active cells could induce follicle synthesis in the mouse’s ovary [123]. Later, in 2012, White and co-workers [124] isolated OSCs from the human ovarian cortex by FACs using the expression of the stem-cell-specific marker VASA. Subsequently, they also documented that these cells were able to induce synthesis of follicles after xenotransplantation into diabetic immunocompromised mice [124]. The identification of these cells contradicts the dogma that only prenatal folliculogenesis is possible in females [46]. However, these cells remained unidentified for a long time, probably because of their very low number, constituting only 0.0145% of the total cell population in mouse ovary that declines gradually with age. Moreover, these cells require a prolonged duration for differentiation in in vitro culture compared to the male counterpart [125]. From a previous study on the isolation of OSCs from old mice and subsequent folliculogenesis following transplantation in young mice [126], it could be speculated that these OSCs could show new hope to those with idiopathic premature ovarian failure. Figure 2 represents the generation of OSCs.

The therapeutic implication of OSCs include restoration of fertility and giving birth to live offspring documented in age-associated infertility and premature ovarian failure in childhood cancer survivors following exposure to chemotherapy [46,127].

### 5.5. Spermatogonial Stem Cell (SSC)

SSCs play a crucial role in maintaining the highly productive complex process known as spermatogenesis in the seminiferous tubule through self-renewal and unlimited differentiation into spermatogonia followed by haploid spermatozoa [128]. These differentiated spermatozoa fertilize oocytes. In mammals, the highly orchestrated spermatogenesis process is categorized into three consecutive phases: (i) proliferative phase (mortification of spermatogonia), (ii) meiotic phase involving recombination of genetic material and (iii) spermiogenesis, which includes the transformation of spermatids into male gametes [129] (Figure 2). Any abnormality in these coordinated phases of spermatogenesis may result in infertility [130]. In an experimental model, it was observed that SSCs are derived from primitive germ cells that travel to the gonadal ridge during embryonic development and then lodge into the seminiferous tubules of adult testicles to help in lifelong sperm generation [131,132]. Only 0.01% of cell populations in the seminiferous tubules of adult mouse testicles are SSCs [133]. In spite of being a very good tool for the treatment of infertility, SSCs are not so frequently used in regenerative medicine because of the very small number of them in testes and difficulty in recognizing them to subsequently isolate and culture them successfully [3]. With the advancement of technology, these SSCs could be isolated and characterized with the help of their unique species-specific identification markers. Hamra et al. [134]) defined SSC-specific molecular markers that could be used to establish a simple way to culture these cells instead of transferring them to testicles. For example, spermatogonia-specific marker Stra8 was used in the isolation of SSCs from mice [135], stage-specific embryonic antigen-1 (SSEA1), β1 and α6 integrins from rat [134], while SSE4 and G-protein coupled receptor 125 (GPR 125) were used for isolation from human [136]. Hermann and his research team documented that SSCs were able to give rise to functional sperm through spermatogenesis and thus could be useful for the successful treatment of some cases of male infertility, especially those induced by chemotherapy. In their study, they transplanted autologous SSCs into 18 adult and 5 prepubertal nonhuman primates (rhesus macaques) made sterile with chemotherapy. Ejaculations from about 75% of adult animals documented the presence of SSCs. Then fertilization of rhesus eggs by allogenic donor SSCs using intracytoplasmic sperm injection gave rise to various stages of embryos. This was ground-breaking work in translational reproductive medicine [137]. Various biomedically active factors, either alone or in combination with others, have been used for in vitro induction of potential stem cells into germ cells: retinoic acid for proper timing in the initiation of meiosis, CYP26 inhibitor for control of meiosis through modulation of the STRA8 gene [138], testosterone for the potential release of stem cell factor to induce germ cell differentiation [139], forskolin to promote proliferation of germ cells [140], and leukemia inhibiting factor (LIF) to keep gonocytes alive [141]. Some studies carried out both in vitro induction of stem cells and subsequent transplantation into male mice that were rendered infertile to prove the ability of SSCs to form colonies within the testes as well as to differentiate into male gametes through highly coordinated spermatogenesis [142].

The main drawback of this cell-based therapy in reproductive medicine is that this procedure may disturb the environment of the testes, resulting in a decline in acceptability of SSC transplantation, giving rise to treatment failure [143].

## 6. Stem Cell Therapy in Some Known Syndromes 

Syndromes leading to infertility are shown in Figure 1 and discussed below:

### 6.1. Asherman Syndrome (AS)

Joseph Asherman, in 1950, was the first to describe a gynecologic disorder in which women presented with amenorrhea following injury to the uterine cavity. This condition was named Asherman Syndrome (AS) after him and is characterized by intrauterine adhesion and partial or complete obliteration of the uterine cavity, giving rise to infertility or recurrent abortion and chronic pelvic pain with amenorrhea [144]. In 90% of cases, AS developed from postpartum curettage [145]. Various diagnostic approaches are available to diagnose this syndrome, including hysterosalpingography (HSG), 3D ultrasonography, hysteroscopy, and magnetic resonance imaging (MRI), with hysteroscopy being the gold standard. Common treatments of infertility resulting from AS include restoration of the uterine cavity through hysteroscopic adhesiolysis and replenishment of normal endometrial lining with hormonal therapy [146,147]. Surgical treatment of AS has been found to be associated with various obstetric complications such as preterm delivery [148]. Hence, the use of stem cell therapy to treat AS has been evaluated.

The use of stem cells derived from bone marrow [149], autologous menstrual blood [83], as well as mesenchymal stem cells [150] for the replenishment of endometrium, has been worked out in various animal models [122] as well as clinical studies [151]. It was observed that endometrial MSCs derived from menstrual blood could improve fertility in an animal model of damaged endometrium [40]. Various studies reported that women with AS attained regular menstruation [152] and endometrial regeneration following transplantation of menstrual-blood-derived stem cells [83].

### 6.2. Premature Ovarian Insufficiency (POI)

POI is an important medical and biological cause of infertility. According to the European Society of Human Reproduction and Embryology (ESHRE) guidelines, diagnostic criteria of POI are “oligo/amenorrhea for at least 4 months and an elevated FSH level >25 IU/l on two occasions >4 weeks apart” [153]. Approximately 1% of women of reproductive age (younger than 40 years) are affected by POI, while in women younger than 30 years and 20 years, the prevalence is around 0.1% and 0.001%, respectively [154]. Hypoestrogenism could affect both the physical and mental wellbeing of the affected person with different magnitudes. With the increase in cancer survivors, the incidence of POI is also increased proportionately. However, in these cases, restoration of fertility has been tried by cryopreservation of oocytes prior to gonadotoxic treatment (Ethics Committee of American Society for Reproductive Medicine, 2013). Several studies have documented that fertility could be preserved in POI cases by ovarian vitrification followed by in vitro activation by stimulation of the AKT pathway followed by re-transplantation [155,156,157]. However, the successful demonstration of ovarian stem cells both in experimental animal and human models might bring revolution in the potential therapy for restoration of infertility in patients with POI [123] (Figure 1).

### 6.3. Polycystic Ovarian Syndrome (PCOS)

PCOS, a gynecological disorder of reproductive-aged women resulting from hormonal imbalance, was initially diagnosed by Rotterdam using two criteria: “(1) presence of high levels of androgens, and (2) ovulatory dysfunction, or polycystic ovaries”. Patients with PCOS commonly present with irregular menstruation, obesity, abnormal hair growth on the body and infertility/subfertility [158]. Ovarian steroidogenesis is regulated by miR-320 by focusing on E2F1 and SF-1 [159]. Hormonal contraceptives and clomiphene citrate are the first-line therapy for restoration of normal menstruation and fertility, respectively [158]. Various experimental models have documented the promising role of stem cells in the alleviation of symptoms of PCOS. MSCs could attenuate DHEA-mediated PCOS in mouse [98] and human theca cells from PCOS patients [160] by suppression of inflammation via the release of anti-inflammatory cytokines. Igboeli and co-workers [159] documented that hMSCs could revert and resume the estrus cycle in a drug-induced mouse model of PCOS [161] (Figure 1).

### 6.4. Endometriosis

Endometriosis is an estrogen-dependent complex disorder affecting women of reproductive age; approximately 10% of women suffer from endometriosis, and approximately 50% of those women with endometriosis have trouble achieving pregnancy [162]. According to The Practice Committee of the American Society for Reproductive Medicine, 2012, the fecundity rate is significantly decreased in couples with endometriosis compared to normal fertile couples [163]. The principal speculations related to the pathogenesis of endometriosis include retrograde menstruation, coelomic metaplasia and metastatic spread, altered immunity, stem cells, and genetics [164]. Endometriosis with its inflammatory response and increased release of cytokines could affect fertility via alteration of ovum formation, gamete transport through impairment of tubal movement, and endometrial development through modulation of the Wnt pathway [165].

A previous study revealed that initial treatment with GnRH agonist followed by IVF with or without ICSI could significantly improve pregnancy rates in infertile couples with endometriosis [166]. However, a later study by Benschop et al. [167] did not find significant improvement in fertility with the initial administration of hormonal therapy [167]. Surgical treatment alone or in combination with medical treatment showed different responses. However, recent experimental evidence in both animal and human models has documented promising results of stem-cell-based therapy for the restoration of fertility in patients with endometriosis [164] (Figure 1).

### 6.5. Azoospermia

Azoospermia, characterized by the absence of mature morphologically normal and functional sperm in the ejaculate, contributes approximately 15% of infertility solely related to male factors [168]. Based on history, detailed physical examination, hormonal assay and genetic testing, azoospermia can be divided into two subtypes: obstructive (OA) and nonobstructive (NOA) azoospermia. OA may be treated by surgical intervention. However, NOA results in testicular failure, which could be due to pathology primarily in the testis or secondary due to decreased release of gonadotropin from the pituitary. In the case of NOA, the success rate of ART by intracytoplasmic injection is very poor because of the difficulty in retrieving functional sperm. Stem cell therapy can bring a ray of hope of having biologically-related offspring to NOA patients [169] (Figure 1).

Spermatogonial stem cells (SSCs), having self-renewal properties, can differentiate into haploid spermatids and finally undergo maturation to spermatozoa and thus might play an important role in promoting sperm production to solve this prevalent cause of male factor infertility [170]. Brinster and Zimmerman [171], for the first time, documented that SSCs could restore fertility in a mice model [171]. Later on, Hermann et al. [137] demonstrated that SSCs injected into the rete testes of pre-pubertal rhesus monkeys undergo a transformation into mature spermatozoa along with the maturation of the monkey; the spermatozoa present in the ejaculate and have fertilization capability [137]. However, SSC transplantation could not be replicated in humans because of challenges associated with the identification of SSCs in the testes, lack of proper culture and preservation protocol for SSCs, and safety concerns for the recipients following transplantation [169]. Other than SSCs, embryonic and adult stem cells were demonstrated to have the ability to be differentiated to germ cells [51] and have the ability to fertilize in animal models [172,173]. However, ethical concerns in the case of hESCs and teratogenicity of iPSCs restricted their therapeutic application [51].

Various clinical trials (NCT02025270, NCT02641769, NCT02414295) have been performed or are underway on injection of bone-marrow-derived MSCs to the rete testis of azoospermia patients to assess hormonal levels as well as testicular size along with sexual potency (see Table 1).

## 7. Clinical Trials

Several clinical trials using various techniques to improve fertility have been completed or are underway. According to ***ClinicalTrials.gov***, as of 7 April 2021, 133 clinical trials on PCOS as a cause of infertility are registered. Out of these, 6 trials are terminated, 55 are already completed, 10 were withdrawn, and recruitment is going on for 10 trials. Seventeen studies have been registered related to stem cells as a therapeutic tool for the treatment of premature ovarian failure. Out of these 17 trials, four are already completed, and one has been withdrawn. Ten trials are registered related to stem cells in azoospermia. Some of these trials related to stem cell therapy for infertility registered to ***ClinicalTrials.gov*** are presented in Table 1 [174,175,176,177,178].

Although stem cell therapy has brought a ray of hope among the medical fraternity, the ethical challenges related to hESC and the safety issues of iPSC, related to unwanted differentiation and oncogenicity and the possibility of MSCs to propagate the tumorous growth, restricted their clinical translation in regenerative and reproductive medicine.

## 8. Future Prospects

### 8.1. Very Small Embryonic Like Stem Cells (VSELs)

VSELs are tiny pluripotent diploid cells (3–5µm) having larger nuclei compared to the cytoplasm and show expression of various stem cell markers, including Oct-4, SSEA, Nanog, and Klf-4, but lack MHC class I, HLA-DR, CD90, CD105, and CD29 [179]. Ratajczak and co-workers first described their existence in various adult animal tissues, including bone marrow [180]. Like other ESCs, in vitro studies documented the capacity of VSELs to be differentiated into all three germ layers. Moreover, their presence in umbilical cord blood and bone marrow has been demonstrated by flow cytometry [181] and differential centrifugation methods [182]. During the initial stage of embryonic development, VSELs are proposed to settle down in different organs, including reproductive organs and remain in a metabolically inactive quiescent state. Following any organ damage, these cells become active to migrate to the injured area to repair it and bring back equilibrium [179]. VSELs reside in the basal layer of seminiferous tubules [183] and surface epithelial cells of ovaries [184]. VSELs are primordial germ cells, undergo self-renewal by irregular cell division, and bring about gonad-specific stem cells such as spermatogonial stem cells (SSCs) and ovarian-specific stem cells [179]. Thus, VSELs play an important role in gametogenesis. 

The discovery of VSELs provides evidence of lifelong renewal of follicles as opposed to the dogma of a fixed number of eggs at the time of delivery and then gradual decline of eggs with age through menopause [185,186]. Parte et al. [184] documented the presence of Oct-4 and SSEA-4 positive special subpopulations of stem cells by confocal microscopy on the surface epithelium of ovaries isolated from various mammals, including humans. However, Zhang et al. [187] contravened the existence of actively dividing germline progenitor cells in adult mouse ovaries. However, the discrepancy in findings observed by Zhang et al. [187] was probably due to the use of a different surface marker, DDX1, which can identify cells of 10–15 µm in size as opposed to VSELs that are smaller in size, i.e., 3–5 µm. Interestingly, Kurkure et al. [188] isolated VSELs by an immune localization method based on the presence of specific marker CD133 and expression of pluripotent and primordial germ cell markers OCT-4 and SSEA-4 as well as STELLA, respectively, from azoospermic testicles of adults who underwent chemotherapy during childhood. They also documented in a mouse model that these VSELs could restore function in the presence of a healthy environment [188].

The discovery of VSELs is a new hope for the collection of autologous pluripotent stem cells from adult reproductive organs, which is less tumorigenic and less immunogenic compared to ESCs. However, more comprehensive research needs to be conducted to identify different technologies for identification, isolation, extensive characterization, and therapeutic implications of VSELs in reproductive medicine. Moreover, the characteristic feature of VSELs to be resistant to chemotherapy would open a new avenue for restoration of fertility in cancer survivors.

### 8.2. Micro RNA and Stem Cell-Based Therapy

MicroRNA (miRNA), small noncoding RNA, plays a crucial role in the expression of genes required for stem cell differentiation via modulation of target mRNA stability and eventually its translation [99]. Upregulation of miR-21 in BMSCs was found to be associated with programmed cell death of granulosa cells as well as an increase in estrogen and decrease in FSH levels in a cyclophosphamide-mediated premature ovarian failure (POF) rat model. The miR-21 in BMSCs contributes to its effect via downregulation of programmed cell death protein 4 (PDCD4) and phosphatase and tensin homolog (PTEN) [189]. BMSC-evolved exosomal miR-644-5p promoted the restoration of ovarian function through its antiapoptotic effect on granulosa cells through the modulation of the p53 signaling pathway [190] as well as PTEN pathway in an experimental animal model of ovarian failure [191].

The miR-10 and miR-146a isolated from membrane-bound extracellular vesicles released by stem cells have been shown to restore ovarian function in a chemotherapy-induced mouse model of POF through the modulation of Bcl-2-like protein 11, interleukin-1 (IL-1) receptor-associated kinase 1, and TNF receptor-associated factor 6 (TRAF6). These findings were validated by repeating the same study after suppression and re-administration of these two micro-RNAs. Of these two miRNAs, miR-10a also showed profound action on the prevention of apoptosis of granulosa cells [192], concluding that microRNAs should also be taken into consideration for facilitating stem cell-based treatment of infertility.

## 9. Conclusions

Infertility creates emotional stress for couples that wish to be parents. Hence, early intervention should be highly beneficial for such couples. Globally, approximately 15% of couples have difficulty conceiving. With the increase in the age of planning to have a baby, the incidence of infertility is increasing. Infertility may result from several factors contributed by a female as well as by a male, or a combination of both. A detailed history of infertility, along with serum hormone levels and semen analysis, could be helpful in determining the major responsible factors. Addressing the etiopathogenesis with ovulation-inducing drugs alone or in combination with IUI might help in most cases. However, some frustrating conditions such as azoospermia and premature ovarian insufficiency might need sperm/ovum donation and assisted reproduction technology. Advanced technologies could solve problems in almost 80% of cases. However, a substantial number of couples cannot conceive even after ART. In such cases, stem cell therapy could bring hope for couples wishing to have their own genetically related offspring. Because of ethical issues and immunologic interference, embryonic stem cell transplantation has lost favor, prompting infertility specialists to explore other stem cell options. Extensive research is being conducted on induced pluripotent stem cells (iPSCs), which have minimal to no ethical concerns and can provide person-specific haploid gametes. Similarly, MSCs, with minimal ethical concerns and derived from various easily available resources, such as bone marrow, adipose tissue, menstrual blood, amnion, amniotic fluid, and placenta, are gaining popularity for their application in reproductive medicine. Many animal and human studies have documented the role of MB-MSCs in endometrial regeneration and restoration of ovarian function. Moreover, knowledge of the contribution of microRNAs in stem cell differentiation would promote a comprehensive understanding of stem cell mechanisms of action for the restoration of fertility. However, more and more large-scale clinical trials are needed to obtain evidence of the safety and efficacy of stem cell-based therapy in the field of human reproduction.

## Figures and Tables

**Table 1:** Clinical trials related stem cell therapy performed or underway for improvement of infertility.

Trial Identifier	Est. # of Subjects	Status	Site	Conditions	Interventions	Outcome of Trial
NCT04706312	12	Not yet recruiting	Nanjing Medical University	Diminished Ovarian Response	Human Amniotic Mesenchymal Stem Cells (Hamscs) Transplantation	No results posted
NCT04676269	40	Recruiting	Indonesia University	Thin Endometrium Infertile Patients	Amnion Bilayer and Stem Cell Combination Therapy	No results posted
NCT03207412	20	Unknown	Chongqing Medical University, China	Premature Ovarian Failure	Human Amniotic Epithelial Cells	No results posted
NCT02696889	3	Active	University of Illinois at Chicago	Primary Ovarian Insufficiency, Low Ovarian Reserve	Autologous Stem Cell Therapy	Report of 2 cases revealed a significant improvement in clinical features related to POI. There was an increase in size as well as estrogen production in the MSC engrafted ovary [174]
NCT02713854	240	Recruiting	The University of Hong Kong	Subfertility	Human Embryonic Stem-Cell-Derived Trophoblastic Spheroid (Bap-Eb) as a Predictive Tool	No results posted
NCT03592849	50	Enrolling by invitation	Nanjing Drum Tower Hospital, China	Infertile Women with Thin Endometrium or Endometrial Scarring	Procedure: Collagen Scaffold Loaded with Umbilical-Cord-Derived Mesenchymal Stem Cells Therapy	No results posted
NCT03166189	46	Completed	D.O. Ott Research Institute of Obstetrics, Gynecology, Russian Federation	Infertility of Uterine Origin Asherman Syndrome	Biological: Bone Marrow-Derived Msc and Hrt	No Results Posted
					Other: Hormonal Replacement Therapy	
NCT02313415	26	Completed	Nanjing Drum Tower Hospital, China	Infertility with Intrauterine Adhesions	Procedure: Umbilical Cord Mesenchymal Stem Cells	Phase 1 trial revealed that transplantation of clinical grade human UC MSC could improve the proliferative and differentiation efficiency of endometrium [175]
NCT02025270	100	Unknown	Al Azhar University, Egypt	Azoospermic Patients	Bone-Marrow-Derived Mesenchymal Stem Cells	No results posted
NCT02641769	50	Recruiting	Stem Cells of Arabia, Amman, Jordan	Non-obstructive Azoospermia	Intratesticular Transplantation of Autologous Stem Cells	No results posted
NCT02414295	1	Completed	Man Clinic for Andrology and male infertilit, Cairo, Egypt	Klinefelter Syndrome Azoospermia	Mesenchymal Stem Cell Injection	No Results Posted
NCT02062931	60	Unknown	Al-Azhar University hospitals, Egypt	Premature Ovarian Failure	Biological: Stem Cell Preparation and Injection	No results posted
NCT02603744	9	Unknown	Royan Institute	Premature Ovarian Failure	Intraovarian Injection of Adipose-Derived Stromal Cells (Adscs)	Intraovarian engrafting of ADSCs were found to be safe and feasible and linked to reduction in FSH level [176]
NCT02204358	30	Unknown	Nanjing University Medical School	Intrauterine Adhesions, Endometrial Dysplasia	Collagen Scaffold Loaded with Autologous Bone	No results posted
					Marrow Stem Cells	
					Testicular Injection of Autologous	
					Human Bone Marrow	
NCT02041910	60	Unknown	Hesham Saeed Elshaer, El-Rayadh Fertility Centre	Azoospermia	Derived Stem Cells	No results posted
NCT02151890	10	Completed	Al Azhar University, Cairo, Egypt	Premature Ovarian Failure	Biological: Stem Cell	No results posted
NCT02372474	112	Completed	Al Azhar University, Cairo, Egypt	Premature Ovarian Failure	Biological: Stem Cell	No results posted
NCT01742533	40	Unknown	Shenzhen People’s Hospital, Shenzhen, Guangdong, China	Premature Ovarian Failure	Biological: Human Umbilical Cord Mesenchymal Stem Cells and Human Cord Blood Mononuclear Cells	No results posted
					Drug: Hormone Replacement Therapy	
NCT03069209	50	Active, not recruiting	Stem Cells Arabia, Amman, Jordan	Premature Ovarian Failure	Biological: Stem Cells	No results posted
NCT00429494	60	Completed	UT MD Anderson Cancer Center, United States	Amenorrhea	Procedure: Hematopoietic Stem Cell Transplantation (Hsct)	Phase II trial revealed that Leuprolide could not preserve ovarian function in HSCT patients [177]
				Premature Ovarian Failure	Drug: Leuprolide Acetate	
				Ovarian Function Insufficiency	Behavioral: Questionnaire	
NCT04009473	100	Enrolling by invitation	Multicenter	Ovarian Failure	Combination Product: SEGOVA Procedure Includes Stem Cell Therapy, Growth Factor, and Platelet Plasma Rich Therapy	No results posted
				Premature Ovarian Failure		
NCT02240823	30	Unknown	Odense University Hospital	Erectile Dysfunction After Prostatectomy	Adipose-Derived Stem Cells (ADMSC)	Intracavernous injection of ADMSC is a safe procedure and resulted in improvement of erectile function [178]
NCT02414308	20	Unknown	Man Clinic for Andrology, Male Infertility, and Sexual Dysfunction	Erectile Dysfunction Peyronie’ Disease	Adipose Tissue Stem Cell Injection	No results posted
NCT02008799	20	Recruiting	Man Clinic for Andrology, Male Infertility, and Sexual Dysfunction	Azoospermia	Intratesticular Artery Injection of Bone Marrow Stem Cell	No result posted
